# Medical students' confidence and competence with prescribing in ST-elevation myocardial infarction: a mixed-methods study

**DOI:** 10.5116/ijme.62c2.c33c

**Published:** 2022-07-29

**Authors:** Teeranan Angkananard, Panida Issarasenarak, Pawita Teerawattananon, Maneekarn Kosulawath, Varunrut Samrejphol, Kamolnetr Okanurak

**Affiliations:** 1 Division of Cardiovascular Medicine, Department of Medicine, Faculty of Medicine, HRH Princess Maha Chakri Sirind-horn Medical Center, Srinakharinwirot University, Nakhon Nayok, Thailand; 2 Medical student of Faculty of Medicine, HRH Princess Maha Chakri Sirindhorn Medical Center, Srinakharinwirot Uni-versity, Nakhon Nayok, Thailand; 3 Department of Social and Environmental Medicine, Faculty of Tropical Medicine, Mahidol University, Bangkok, Thailand

**Keywords:** Confidence, prescription, antiplatelets, fibrinolytic agents, STEMI

## Abstract

**Objectives:**

To explore factors associated with prescribing confidence and competence of final-year medical students for prescribing antiplatelet and fibrinolytic agents in ST-segment elevation myocardial infarction (STEMI).

**Methods:**

The study was conducted among final-year medical students with a triangular convergent mixed-methods approach. First, an online survey was conducted using a voluntary sampling method with concurrent in-depth interviews performed. The survey data was analysed using descriptive statistics and paired t-tests, while survey factors were compared using the chi-squared or Fisher's exact test. The interview data were coded and analysed thematically. The relations between the qualitative and quantitative findings were finally described.

**Results:**

Totally 92 validly replied to the questionnaire, and 20 participated in the interviews. The quantitative analysis indicated that they had high competence in the diagnosis of STEMI and prescribing antiplatelet and fibrinolytic agents. The mean confidence score of prescribing for both was medium and was significantly lower in fibrinolytic agents. (M=3.3, SD=1.1 vs. M=2.8, SD=1.0, t_(91)_=5.39, p<0.01). Their experience, knowledge, and mentoring were accounted for, considering the prescribing confidence factors in both approaches. Besides, providing guidelines and standing orders were derived from the interview data.

**Conclusions:**

This study has demonstrated that final-year medical students have a high ability to diagnose and prescribe essential medications in STEMI but tend to have low confidence in prescribing fibrinolytic agents. Experiential learning, mentorship and providing guidelines can help them, especially in emergency settings to prescribe confidently and safely. Further multicenter studies on undergraduate and graduate medical students’ confidence and perspective of prescribing are required, especially for high-alert medications.

## Introduction

ST-segment elevation myocardial infarction (STEMI), which is classified as an acute coronary syndrome, is one of the principal causes of death and disability.^1^ Nearly 18 million people died from cardiovascular diseases in 2019, of which 85% died from heart attack and stroke.^2^ Furthermore, it has been predicted that cardiovascular diseases will cause more than 22 million deaths by 2030.^3^ In the United States, an American has an acute myocardial infarction (AMI) approximately every 40 seconds.^3^ AMI is thus a time-sensitive condition, and proper emergency management is also essential. Therefore, physicians need to be able to diagnose and manage patients with AMI promptly and correctly in any clinical setting, particularly during emergency visits. Updated guidelines^4^^,^^5^ for the management of AMI has been published regularly, although the final decisions for patients’ management with AMI must be made by physicians individually, following an appropriate diagnosis, investigation and prescription.

Dual antiplatelet therapy comprises the combination of aspirin and an oral inhibitor of platelet P2Y12 receptor for adenosine 5’-diphosphate (clopidogrel, ticagrelor and prasugrel), which is indicated for the first period after AMI or percutaneous coronary intervention (PCI).^6^ They can reduce the risk of stent thrombosis, and spontaneous MI, which are associated with high mortality rates. However, it is also related to increased bleeding risk. Similarly, fibrinolytic agents (streptokinase, alteplase, reteplase, and tenecteplase) offer an alternative therapy for reperfusion and are used in pharmaco-invasive approaches with PCI in cases of STEMI. There is a propensity for critical bleeding, particularly the risk of intracranial hemorrhage,^7^ and thus it should be prescribed carefully and in a timely fashion. Inappropriate and delayed prescribing of antiplatelets and fibrinolytic agents are the major modifiable contributors to the outcome of patients with STEMI. Due to the limitations of PCI centres, 20% of Thai patients with STEMI have received thrombolysis for reperfusion and still experienced a higher mortality rate than those receiving PCI.^8^ Thus, patient safety while confidently and accurately prescribing antiplatelet and fibrinolytic agents is essential for patients’ outcomes.

Worldwide medical curricula include clinical pharmacology and prescribing medications for medical students to improve their prescribing competencies in clinical practice. Currently, there is little evidence of instruments to assess the confidence and knowledge of medical students for prescribing antiplatelet and fibrinolytic agents in STEMI when compared to other diseases. Hence, this study aimed to investigate the factors associated with the self-reported confidence of final-year medical students and their competence in accurately prescribing antiplatelet and fibrinolytic agents for patients with STEMI.

## Methods

### Study design

**Figure 1:**
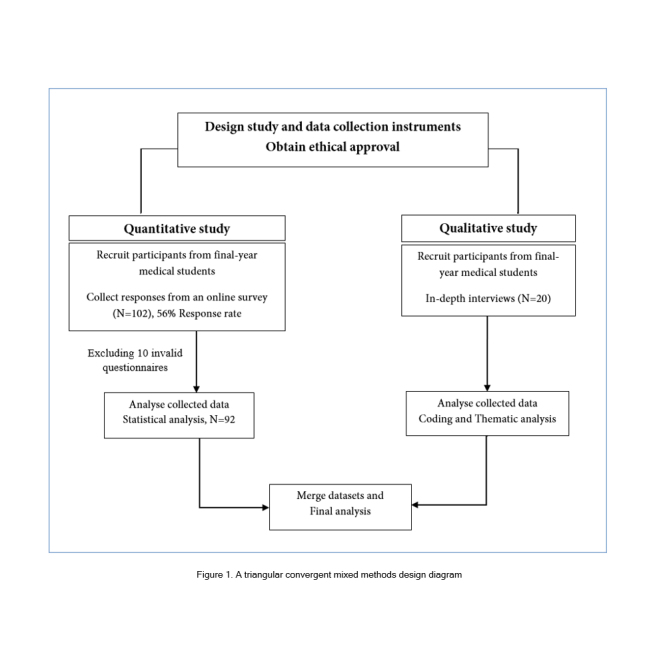
A triangular convergent mixed methods design diagram

A triangular convergent mixed methods design was applied to obtain a more thorough understanding of influencing factors for prescribing confidence of final-year medical students in the emergency setting of STEMI. An online survey using the voluntary sampling method and in-depth interviews using purposive sampling were performed simultaneously, analysed separately and then integrated into the ﬁnal analyses (Figure 1).^9^^-^^11^ Both quantitative and qualitative data were collected during the same period of study and targeted to achieve the objective of the study and facilitate triangulation and credibility. Qualitative methods were required due to the limited data and comprehensive appreciation of their confidence and competence for a prescription for a case with STEMI. To enrich the interpretation of findings, the relationships between the qualitative and quantitative results were described.

The study was conducted between November 1, 2020, and April 30, 2021, in one single center to minimise confounding effects from different environments, clinical rotation experience, and basic medical training programs. Ethical approval was considered by the Institutional Review Board of Srinakharinwirot University, Thailand (SWUEC-332/2563E). Written informed consent was obtained from all final-year medical students who participated in the study. Participation in the study was free, and all data were presented anonymously.

#### The quantitative method

##### Survey instrument

A Google Form self-administered questionnaire focused on the details of their accuracy for diagnosis and confidence of prescribing essential medications (antiplatelets and fibrinolytic agents) for a clinical case of STEMI according to the standard guideline.^4^^,^^5^ The survey items were initially developed in English and then translated into Thai, which were independently reviewed by two experts (T.A. and K.O.), who are specialised in cardiology, and public health, to ensure that the item questions adequately met and were all relevant to the aim of the study. After that, there was one meeting with the research team to discuss the survey items. To ensure the questions were understandable, accurate, structured, and cohesive, those which did not reach consensus were modified or discarded.

Before sending out the survey, preliminary pilot testing of the questionnaire was performed with 25 final-year medical students, and the questions were revised upon reviewing the results of that pilot test. The final draft of the questionnaire after pilot testing consisted of 23-item open-and closed-ended questions and divided into three categories: 1) demographics, 2) knowledge, and 3) prescribing confidence. The demographic section included gender, age and completed clinical practice in the internal medicine department. The knowledge section consisted of 8 questions comprising three multiple-choice questions, two know/do not know, and three free text tests. Knowledge data included the correct diagnosis of a case presented with STEMI, and prescribing knowledge of types and dosages of antiplatelets and fibrinolytic agents. In the section on prescribing confidence, the students were asked to rank their confidence in prescribing both medications by themselves as a 5-point Likert scale^12^ (1=very unsure; 2=slightly confident; 3=somewhat confident; 4=fairly confident; 5=very confident). That scale included a midpoint and could be used as an interval scale for statistical analysis purposes. The respondents were not pressured to agree or disagree, which could decrease the number of nonresponses.^13^ In addition, they were requested to comment on two free-text answers, and 8 provided possible barriers to their confidence in the prescription. Test score reliability of their attitude to factors affecting prescribing confidence of antiplatelets and fibrinolytic agents were acceptable (Cronbach alpha^14^=0.83 and 0.82, respectively).

#### Respondents

All 175 final-year medical students of the academic year of 2020, Faculty of Medicine of Srinakharinwirot University, Thailand, were invited to answer a Google Form questionnaire through a QR code. Google Forms requires respondents to be signed into a Google account of Srinakharinwirot University to complete the survey, which could prevent multiple entries from each individual. Their email addresses were not collected to maintain anonymousness. To maximise the response rate, all respondents could freely complete the questionnaire at any time by scanning QR codes or using the URL link of Google Form on their mobile phones. A prenotification note by an academic officer who was not involved in this study was sent to the students’ LINE Group to increase the participation rate.^15^

Totally 102 final-year medical students replied to the survey (response rate, 58.3%). Data were gathered from a final sample of 92 respondents after excluding ten invalid questionnaires. Table 1 shows their characteristics and the results obtained from the questionnaire survey responses, of whom 56 (60.9%) were female and 36 (39.1%) were male. The majority of the respondents, 84 (91.3%), had interned in medical wards.

### Data analysis

Missing data are unavoidable when data are collected using questionnaires. A web-based self-administered questionnaire was used in this study to diminish respondents’ stress and the item nonresponse as a result of the respondent’s mistakes or refusals. Some items in the questionnaire tested recall and could be answered ‘do-not-know’, for instance, a question about the dosage of fibrinolytic agents. Therefore, those data can be missing completely at random, and a complete case analysis was used to handle it.^16^

**Table 1:** Questionnaire survey responses (N=92)

Variables		N (%)
Gender		
	Female	56 (60.9)
	Male	36 (39.1)
Interning in medical ward		
	Yes	84 (91.3)
	No	8 (8.7)
Correctly diagnosis of STEMI		
	Yes	71 (77.2)
	No	13 (14.1)
	Unsure	8 (8.7)
Correctly prescribe antiplatelets		
	Type	83 (90.2)
	Dosage	64 (71.9)
Correctly prescribe fibrinolytic agents (n=86)		
	Type^*^	74 (86.0)
	Dosage^*^	25 (80.6)
Confident score to prescribe antiplatelets		3.3 (1.1)^†^
	1=Not at all	7 (7.6)
	2=Slightly	15 (16.3)
	3=Somewhat	29 (31.5)
	4=Fairly	28 (30.4)
	5=Completely	13 (14.1)
Confident score to prescribe fibrinolytic agents		2.8 (1.0)^+^
	1=Not at all	14 (15.2)
	2=Slightly	19 (20.7)
	3=Somewhat	35 (38.0)
	4=Fairly	23 (25.0)
	5=Completely	1 (1.1)

STEMI, ST-segment elevation myocardial infarction; *Missing data: type of fibrinolytic agents=12, dose of fibrinolytic agents=57;† Data are reported by mean (SD)

Categorical variables are shown as frequency and percentages. The levels of confidence for prescribing antiplatelet and fibrinolytic agents were scored from 1 to 5 according to the 5-point Likert scale and classified into two groups: low- and high confidence based on their means.^13^ The Likert scale responses of their confidence in prescription were analysed using parametric statistics^17^ and were provided as mean (M) with standard deviation (SD) and compared with paired t-test.

The influencing factors of their confidence in prescribing both medications were expressed as frequency and percentages in case they also agreed. Those were compared between low- and high-confident groups using a chi-squared test (χ^2^) or Fisher's exact test. The p-values were two-sided, and a value of less than 0.05 was considered a significant difference. Data were analysed using STATA version 16.1.

### The qualitative method

During survey data collection, in-depth interviews were conducted in the same period. Purposive sampling was used to recruit the study participants. For inclusion, all participants (25-27 years, n=20) who were final-year medical students and graduates in the academic year of 2020 at the time of the interview and agreed to the audio recording of interview sessions were enrolled. All participants were invited to an in-depth interview during their responses to an online survey. Therefore, they were able to participate and verify in both approaches, and remuneration was not given. The interview concentrated on the interviewees’ experience, self-reported confidence in diagnosis and prescription, and their attitude to barriers to prescribing antiplatelet and fibrinolytic agents for patients with STEMI.

Four trained female study investigators (P.I., P.T., M.K., and V.S.) conducted interviews face-to-face (n=15) in private rooms located within the hospital area or by telephone (n=5) using a semi-structured interview list which consisted of open- and closed-ended questions, initially developed in English and then translated into Thai (Appendix). Each interview was individually operated by one study staff who was randomly assigned to five participants. The written informed consents were collected, and interview data were audio-recorded by one mobile phone, which was only used in this study. Study numbers without personal identifiers were uniquely assigned for each subject. All participants who also responded to the online survey included 10 (50%) female and 10 (50%) male students with a mean age of 26.0 (SD=0.8) years. The median time of interviews lasted 18.02 (IQR:16.71- 21.35) minutes, and they were conducted in the Thai language.

### Data collection and analysis

Initially, all four interviewers were trained, reviewed and elucidated on the probing technique for the first five interviews. Then they applied to collect insight information from the interviewees. Conversations were transcribed verbatim in Thai and translated to English after checking by T.A. and they were not returned to participants for comment. All transcription of the data was performed manually without the use of a database.

An inductive thematic analysis was performed following steps documented by Braun and Clarke.^18^ To understand the factors affecting their confidence in prescribing antiplatelet and fibrinolytic medications, the interview transcripts were reviewed, and initial codes were generated, with searching, reviewing and naming for themes. Saturation of themes was achieved during data collection by all interviewers in discussion together. All discrepancies were resolved by consensus with the author (T.A.).

### Data combination and synthesis method

To evaluate in what ways the results of prescribing confidence factors converged or diverged, the attitude of confidence factors provided and free text responses in the questionnaires were compared with themes derived from the qualitative interviews.

**Table 2:** Differences in survey responses of low versus high confidence groups stratified by prescription medication

Characteristics	Antiplatelets			Fibrinolytic agents		
	Low Confidence (N=51)	High Confidence (N=41)	p-value	Low Confidence (N=33)	High Confidence (N=59)	p-value
	N (%)	N (%)		N (%)	N (%)	
Gender						
Female	28 (54.9)	28 (68.3)	0.19	20 (60.6)	36 (61.0)	0.97
Male	23 (45.1)	13 (31.7)		13 (39.4)	23 (38.9)	
Medical ward practice	44 (86.3)	40 (97.6)	0.07	29 (87.9)	55 (93.2)	0.45
Correct diagnosis of STEMI	36 (70.6)^*^	35 (85.4)^*^	0.24	23 (69.7)	48 (81.4)	0.33
Correct prescribing type of medication	44 (86.3)	39 (95.1)	0.29	29 (87.9)^†^	46 (77.9)^†^	0.28
Correct prescribing dosage of medication	29 (56.8)^*^^*^	35 (85.4)	0.007	6 (18.2)^‡^	20 (33.9)^‡^	0.25
Attitudes of confident factors						
Confidence in correct diagnosis of STEMI	28 (54.9)	33 (80.5)	0.01	17 (51.5)	44 (74.6)	0.03
Experience for prescription	39 (76.5)	23 (56.1)	0.04	28 (84.8)	36 (61.0)	0.02
Teaching by instructors and/or residents	22 (43.1)	24 (58.5)	0.14	13 (39.4)	37 (62.7)	0.03
Supervision during prescription	33 (64.7)	25 (60.9)	0.71	22 (66.7)	39 (66.1)	0.96

STEMI: ST-segment elevation myocardial infarction; *Missing data: low-confident = 5, high-confident = 3; **Missing data: low-confident = 5; †Missing data: low-confident = 3, high-confident= 9; ‡Missing data: low-confident= 26, high-confident= 35

## Results

### Quantitative results

Seventy-one respondents (77.2%) correctly diagnosed a case scenario of STEMI, while 83 (90.2%) and 64 (71.9%) respondents accurately prescribed the type and dosage of antiplatelets, respectively (Table 1). Nearly all (n=86, 93.5%) responded to recognising the fibrinolytic agents in treatment STEMI, although some data collected on type and dosage of prescribing fibrinolytic agents were missing, 12 (13.9%) and 57 (66.3%), respectively. Seventy-four (86.0%) and 25 (80.6%) of them could correctly answer the type and dosage of fibrinolytic agents, respectively. The self-reported confidence score of both prescriptions varied in range from 1 to 5, and median of both of them was 3.0. The mean self-reported confidence score of prescribing antiplatelet and fibrinolytic agents were medium, in which the former was significantly higher than the latter (M=3.3, SD=1.1 vs M=2.8, SD=1.0, t _(91)_ =5.39, p<0.01). The very confident score (score=5) was rarely reported for both antiplatelets (n=13, 14.1%) and fibrinolytic agents (n=1, 1.1%) prescriptions. Their attitude of confidence factors in prescribing antiplatelet and fibrinolytic agents were ranked as follows: experience with prescription, 62(67.4%) vs 64(69.6%); confidence in correctly diagnosis of STEMI, 61(66.3%) vs 61(66.3%); supervision during prescription, 58(63.0%) vs 61(66.3%); and teaching by attending physicians and/or residents, 46(50.0%) vs 50(54.3%), respectively (data not shown).

Table 2 demonstrates no significant difference between low- and high-confident groups of prescribing antiplatelets and fibrinolytic agents with regard to their gender, medical ward practice, their ability of diagnosis STEMI and prescribing type of those medications. Nonetheless, their competence for accurate prescribing dosage of antiplatelets were notably higher in high-confident group [low- vs. high-: 29 (56.8%) vs. 35 (85.4%), χ^2^_(1, N =92)_= 9.85, p=0.007], while there was no significant difference for prescribing type and dosage of fibrinolytic agents [low- vs. high-: 6 (18.2%) vs. 20 (33.9%), p=0.25, N 35, Fisher's Exact Test]. By comparing between low- and high-confident groups of their perspectives of prescribing confidence in both medications, previous experience of prescription was significantly recognised in the former, while the correct diagnosis of STEMI was considerably reported in the latter. Teaching and supervision during prescription by their instructors and/or residents were additionally listed in which teaching in class was considerably reported in high-confident students for prescribing fibrinolytic medications [low- vs high-: 13 (39.4%) vs 37 (62.7%), p=0.03, N 92, Fisher's Exact Test]. However, supervision during the prescription of both medications were not differently reported as for their confidence factors between the two groups.

### Qualitative results

Table 3 illustrates the characteristics of 20 final-year medical students and their interview data. Twelve of them had trained in medical wards and encountered STEMI at medical wards with a median case of 5 (IQR: 4-15), while 11 of them had experienced 1-2 cases of STEMI presented at the emergency room. When compared with prescribing antiplatelets, they tended to have lower confidence in prescribing fibrinolytic agents.

**Table 3:** Qualitative interview data (N=20)

No.	Gender	Age Yrs	Duration of interview (min)	Mode of interview	Medical ward practice	No. STEMI cases (ER)	No. STEMI cases (ward)	No. STEMI cases prescription	Level of confidence to diagnose STEMI	Level of confidence to prescribe antiplatelet	Level of confidence to prescribe fibrinolytic agents	No. of cases to boost confidence	Confidence Builders
1	Male	26	12’45’’	Face-to-Face	Yes	0	9	0	high	high	low	5	Mentoring
2	Male	26	16’37’’	Face-to-Face	Yes	0	2	0	medium	medium	low	5	Mentoring/Experience
3	Male	27	16’48’’	Face-to-Face	Yes	0	15	n/a	medium	medium	low	20	Mentoring/Experience
4	Male	25	21’06”	Face-to-Face	No	0	7	0	high	medium	medium	2	Mentoring/Experience
5	Male	26	21’36’’	Face-to-Face	No	0	3	0	low	low	low	5	Mentoring/Experience
6	Male	25	19’00”	Face-to-Face	No	2	0	2	medium	medium	low	10	Mentoring/Experience
7	Female	26	18’00”	Face-to-Face	No	1	4	0	low	low	low	7	Mentoring/Experience
8	Male	26	50’00”	Face-to-Face	Yes	2	4	0	medium	high	high	4	Mentoring
9	Male	27	17’00”	Face-to-Face	Yes	0	6	1	medium	low	low	3	Mentoring/Experience
10	Male	25	17’00”	Face-to-Face	No	1	10	0	medium	medium	low	3	Mentoring /Experience
11	Female	25	15’03”	Tele-phone	Yes	0	20	n/a	high	high	high	2	Mentoring/Practice of reading ECG
12	Female	27	17’29”	Tele-phone	No	1	n/a	0	medium	medium	low	10	Mentoring/Practice of reading ECG
13	Female	26	18’02”	Tele-phone	No	1	5	0	medium	medium	low	10	Mentoring/Self-directed learning/Experience
14	Female	26	21’02”	Tele-phone	Yes	1	5	n/a	medium	high	high	3	Mentoring/Self-directed learning
15	Female	26	14’39”	Tele-phone	Yes	1	25	3	medium	high	medium	3	Mentoring/Providing guideline
16	Female	28	20’01”	Face-to-Face	Yes	0	5	0	n/a	medium	medium	n/a	Teaching in class/ Experience
17	Female	26	15’37”	Face-to-Face	Yes	1	20	n/a	n/a	high	medium	4	Teaching in class/ Experience
18	Female	26	25’20”	Face-to-Face	Yes	1	5	n/a	n/a	low	low	n/a	Mentoring/Teaching in class/Experience
19	Male	25	27’14”	Face-to-Face	Yes	1	30	n/a	n/a	high	medium	3	Teaching in class/ Experience
20	Female	26	22’57”	Face-to-Face	No	0	5	0	medium	medium	low	5	Mentoring/Providing guideline/Case-based study

STEMI: ST-segment elevation myocardial infarction; ER, emergency room; n/a, not applicable

**Table 4:** Qualitative confidence domains, themes and corresponding subcategories

Domains	Themes	Subcategories	Quotations of participants
Confidence Influencers	Experience	Own experience	“I’m still anxious because it's my first attempt to manage these on my own fully.”
			“I'm quite nervous since I haven't prescribed it very often.”
			“I'm uncertain as I haven't yet attended a medical ward.”
	Knowledge	Recognition (dosage, side effects and adverse events of medication)	“I'm concerned about complications in case we do not know or recall precisely the cautions of those medications.”
			“I’m anxious about the safety of my patients if I can't remember their dosages accurately.”
		Correct diagnosis of STEMI	“I will be confident if the case and ECG are evidently obvious. If it's not apparent, I will priorly consult the residents or our staff.”
	Providing guidelines	Standing order provided	“Whereas I somewhat know, but I’m not certain, I’d rather prescribe medication as stated in the standing order.”
	Mentoring	Mentorship	“I’m quite confident because I have already seen and managed two cases under the supervision of the residents.”
Confidence Builders	Experience	Own experience	“Previously, I used to prescribe this medication before, so I’m pretty sure to do it again.”
			“I’m studying in a medical ward where I have been practising reading ECGs with the STEMI cases. Therefore, I’m quite sure when I see the cases fit with their ECG.”
	Knowledge	Self-directed learning	“Practice reading ECG accurately is essential.”
			“Self-review of the lessons would help me remember better.”
			“I have to memorise the drug dosage or make a summary note so that I can easily find it later.”
		Teaching in class	“Case-based teaching helps me in managing the patient with STEMI by myself.”
	Providing guidelines	Guidelines provided	“I feel more confident when I follow the guideline of STEMI.”
	Mentoring	Mentorship	“Should ask/ consult the attending physicians or residents to confirm my case.”
			“It would be helpful if someone could observe and assist me while I’m reading the ECG of STEMI cases.”
			“In my opinion, mentoring and approval of my diagnosis of STEMI and prescription order of essential medications by teachers and/or residents could build up my confidence in prescription.”

Thematic analysis demonstrated two domains (influencers and builders of confidence) and four themes: (1) experience, (2) knowledge, (3) providing guidelines and (4) mentoring (Table 4). They were derived from the theme codes of recall, own experience, anxiety, fear, teaching, peer-review, approval, consult, clinical practice, guidelines, standing order, and electrocardiography (ECG). Mentoring was frequently mentioned as their confidence builder for the prescription. The details of the four themes are:

(1)  Experience refers to their own experience with the real cases in aspects of diagnosis of STEMI and prescription.

Own experience with the real cases was recognised as having an impact on their confidence in prescribing antiplatelet and fibrinolytic agents. The following statements are offered verbatim as examples:

“I’m still anxious because it's my first attempt to fully manage these on my own.” (No.6, Female, 25 years)

“I’m studying in a medical ward where I have been practising reading ECGs with the STEMI cases. Therefore, I’m quite sure when I see the cases fit with their ECG.” (No.12, Female, 27 years)

(2)  Knowledge relates to recall, teaching in class, and self-directed learning. This theme is defined by the ability of all the participants to diagnose cases with STEMI correctly and recognise medication regarding the type, dosage, adverse effects, and cautions to use. One participant said:

“I’m anxious about the safety of my patients if I can't remember their dosages accurately.” (No.9, male, 27 years)

To increase self-confidence in accurately prescribing both medications, self-directed learning which was related to reading, review and practice, teaching in class, and searching on the internet, were quoted. They also recognised the importance of accurately reading an ECG for diagnosis of STEMI and case-based teaching. Some mentioned:

“Case-based teaching helps me in managing the patient with STEMI by myself” (No. 8, male, 26 years)

“Practice reading ECGs accurately is essential.” (No.10, male, 25 years)

“Self-review of the lessons would help me remember better.” (No.13, Female, 26 years)

(3)  Providing guidelines was themed from adhering to clinical practice guidelines and standing order. It was reported as both a confidence influencer and builder on their prescriptions. One of them stated:

“Whereas I somewhat know, but I’m not certain, I’d rather prescribe medication as stated in the standing order.” (No. 14, Female, 26 years)

(4)  Mentoring was quoted by approval, consultation, and ECG recognition of cases with STEMI. Some mentioned that to increase self-confidence in accurately prescribing both medications, they needed to be supervised by their instructor and/or a senior doctor during their management and prescription. Some of them described:

“In my opinion, mentoring and approval of my diagnosis of STEMI and prescription order of essential medications by teachers and/or residents could build up my confidence in prescription.” (No. 2, male, 26 years)

“In life-threatening cases such as STEMI, I would like my staff come and see with me” (No.8, male, 26 years)

“It would be helpful if someone could observe and assist me while I’m reading the ECG of STEMI.” (No.11, Female, 25 years)

### Combination results

The attitude of confidence factors for prescribing essential medications in STEMI of final-year medical students, which was derived from surveys and interviews, converged. Their experience, knowledge and mentoring were reported in both approaches when only providing guidelines was complementarily found from the qualitative method.

## Discussion

This study was initiated to examine the factors associated with the self-reported confidence of final-year medical students and their proficiency in prescribing vital medications for patients with STEMI.

The quantitative analysis has revealed that they have high competence in diagnosis and prescribing antiplatelet and fibrinolytic agents. However, self-reported confidence for prescribing them were both medium, and significantly lower for prescribing fibrinolytic agents. The level of confidence was not apparently correlated with their ability for accurate diagnosis, but considerably associated with their precise prescription, particularly of antiplatelets. This may indicate the importance of prescribing confidence in the accurate and safe prescription of STEMI. With regard to their confidence factors for prescription, prior prescribing experience, and their knowledge (awareness of correct diagnosis of STEMI and teaching in class) were substantially concerned in the low-and high-confident students, respectively.

In addition, the qualitative results indicated experience, knowledge, providing direction and mentoring related to medical students’ prescribing confidence of STEMI and were considered as their confidence influencers and builders. Providing guidelines and standing order were complementarily discovered from the interview data. The results of both approaches were similar with regard to their attitude toward prescribing confidence factors. Those emphasised the important role of all four themed factors in enhancing medical students’ confidence and competence in prescribing essential medication in the emergency management of STEMI.

Prior evidence reported that final-year medical students could prescribe medication safely and effectively.^19^^,^^20^ The major themes are affecting prescribing competence and confidence of medical and pharmacy students were lacking self-awareness, knowledge, and experience,^20^^,^^21^ which result was similar to the findings in this study. A notable correlation between the proportion of accurate answers and confidence in clinical cases and treatment related to cardiology has been reported.^21^ Additionally, there was no difference in clinical knowledge between undergraduate and graduate medical degree students.^21^^,^^22^ The mean score of self-reported confidence for prescription in this study was intermediate, while the proportion of correct answers for prescription was quite high. Possible explanations for this inconsistency are their low authority and assertiveness in taking care of and prescribing drugs for emergency cases. As described earlier, STEMI is a time-sensitive condition and one of the main causes of global morbidity and mortality. Thus, prompt diagnosis and early management by first-contact medical personnel are mandatory. This high degree of responsibility would mainly belong to a cardiologist or a senior physician. Therefore, final medical students may have an insignificant role, which leads to low self-perceived confidence in prescribing essential medications for patients with STEMI. This qualitative study confirms that having clinical case practices and mentoring were possible factors influencing their confidence. Moreover, lack of knowledge, recognition and training for diagnosis and treatment of STEMI were revealed as barriers to their immediate decision and management.

Furthermore, the findings of this demonstrated that the mean confidence score was significantly reported higher for prescribing antiplatelets than fibrinolytic agents. This could be explained by infrequent use, a substantial number of cautions, and the possibility of serious complications correlated to the prescription of the latter. This is supported by the qualitative results of this study, which showed that recalling the drug dosage, and anxiety of complication were considered as obstacles for their prescription.

This study had several strengths. This is the first analysis of the self-reported confidence and competence of medical students to prescribe essential medications in STEMI. This study found the Dunning–Kruger effect^23^ in medical students who may have a cognitive bias of illusory inferiority to underrate their abilities for their prescription. This study also explored the possible factors that could improve their self-confidence for prescribing those medications. They required mentorship, particularly from senior residents and/or attending staff, to confirm their ECG interpretation, diagnosis of STEMI, and writing orders, including prescriptions. In addition, they realised that self-directed learning, adhering to the clinical practice guidelines or following the providing standing order of STEMI management could increase their confidence for self-prescription.

This study had several limitations. First, it was conducted in a single medical university on a small number of participants, which may have an introduced bias and not be representative of all Thai medical students. Second, the study participants were solely a group of final-year medical students, which may cause missing perspectives from lower and higher experienced groups, such as fourth-year medical students, junior doctors and residents. Third, the questionnaire in this study was developed to evaluate both confident and competent factors, so previously established self-esteem questionnaires were not applied in this study. Fourth, there was plenty of missing data for responding to the dosage of fibrinolytic agents, which was handled by complete case analysis. This could reduce the study power and may be unable to differentiate correct answers of their dosages between low- and high-confident groups. Lastly, some confounders, such as their families, personality, childhood religion, and grade point average, which may contribute to their self-esteem, were not examined in this study because of confidentiality, the limited time and skills of the interviewers.

The findings from this study have revealed the possible barriers to the final-year medical students' confidence in prescribing essential medications in cases with STEMI. According to the results of this study, experience-based learning^24^ and mentorship may boost undergraduate medical students’ prescribing confidence and competence, especially in emergency situations. In addition, integrating pharmacological training, as well as case-^25^ and simulation-based learning^26^ would be helpful to prescribe safely and confidently. Further multicenter studies on prescribing confidence and attitude, especially of high alert medications should be investigated in both undergraduate and graduate medical students.

## Conclusions

This study has demonstrated that final-year medical students have a high ability to diagnose and prescribe antiplatelet and fibrinolytic agents in STEMI but tend to have low confidence in prescribing fibrinolytic agents. Experiential learning, mentorship and providing guidelines can help them, especially in emergency settings to prescribe confidently and safely. Further multicenter studies on undergraduate and graduate medical students’ confidence and perspective of prescription are required, especially for high-alert medications.

### Acknowledgement

The authors would like to thank Stephen John Pinder (native speaker) and Nattakrit Tongpoonsakdi for conducting a comprehensive English language review on this manuscript, and all last-year medical students of Faculty of Medicine, Srinakharinwirot University and Maneerat, Pinngernor, our academic officer, for their valuable support of this research work.

### Conflict of Interest

The authors declare that they have no conflict of interest.

## Supplementary material

Supplementary file 1 Appendix. Medical students' confidence and competence with prescribing in ST-elevation myocardial infarction: a mixed-methods study: In-depth interview guide
