# Supplementary material for: Medical students' confidence and competence with prescribing in ST-elevation myocardial infarction: a mixed-methods study
Source: Int J Med Educ. 2022 Jul 29;13:187–97. doi: 10.5116/ijme.62c2.c33c (PMC9911138; doi:10.5116/ijme.62c2.c33c)
Supplement: Supplementary file 1 — Appendix. Medical students' confidence and competence with prescribing in ST-elevation myocardial infarction: a mixed-methods study: In-depth interview guide [file ijme-13-187-S1.pdf]

## Appendix A

Medical students' confidence and competence with prescribing in ST-elevation myocardial infarction: a mixed-methods study: In-depth interview guide

| Main topics | Medical students' factors for prescription in patient with STEMI |
|---|---|
| 1) General | • Have you already trained in a medical ward?<br>• What kind of learning in year 6 do you like and why?<br>• Which ward do you like most?<br>• Which subspecialty would you like to study after graduation? |
| 2) STEMI Cases | • How many cases of STEMI did you encounter at the emergency room (meet them first)?<br>• How many cases of stable STEMI did you encounter or take care of at the medical ward?<br>• How do you feel when you come across a case like this?<br>• How confident are you in the diagnosis of STEMI? |
| 3) Prescription | • How many cases of STEMI did you prescribe medication by yourself?<br>• How much do you understand the disease and treatment of STEMI?<br>• How much do you understand about the medication that you/ resident(s)/ your teacher(s) prescribe?<br>• Do you know the dosages, side effects and cautions of antiplatelets?<br>• Do you know the dosages, side effects and cautions of fibrinolytic agents?<br>• What do you think about bedside teaching or case-based learning of STEMI cases for gaining confidence for prescription?<br>• If you are not confident in your prescription, which one will help you to be confident?<br>• How many cases of STEMI are to be encountered that you think is enough for your confidence in prescription?<br>• To boost your confidence in prescribing, what do you think about the provided standing order or guideline? |
| 4) Comment | • Is there anything else you would like to share about your confidence in prescription in patients with STEMI? |
